# Detection of metallo-beta lactamases and oxacillinase genes in carbapenem-resistant *Acinetobacter baumannii* strains isolated in Morocco

**DOI:** 10.11604/pamj.2021.40.210.28663

**Published:** 2021-12-07

**Authors:** Abdelhamid Massik, Lahbib Hibaoui, Btissam Arhoune, Ghita Yahyaoui, Bouchra Oumokhtar, Mustapha Mahmoud

**Affiliations:** 1 Laboratory of Microbiology and Molecular Biology, University Hospital Center (CHU) Hassan II, Fez, Morocco,; 2 Biomedical and Translational Research Laboratory, Faculty of Medicine and Pharmacy, Sidi Mohammed Ben Abdellah University, Fez, Morocco,; 3 Laboratory of Microbiology and Molecular biology, Faculty of Medicine and Pharmacy, Sidi Mohammed Ben Abdellah University, Fez, Morocco

**Keywords:** *Acinetobacter baumannii*, metallo-beta lactamases, oxacillinases

## Abstract

**Introduction:**

resistance to carbapenem is widespread among Acinetobacter baumannii (A. baumannii) strains. Metallo-beta lactamases enzymes (MBL) are responsible for carbapenem resistance, as are oxacillinases (OXA). In recent years, MBL producing carbapenem-resistant strains have been reported in the world and Morocco at increasing rates. Our study aimed to investigate the presence of carbapenemases in acinetobacter strains isolated from hospitalized patients in CHU Fez.

**Methods:**

a total of 58 imipenem-resistant A. baumannii strains isolated from clinical samples were investigated. The presence of MBL was described phenotypically by the double-disk synergy test (DDST), MBL E-test, and modified Hodge test. The bla_IMP_, bla_VIM_, genes, and bla_OXA-23_, bla_OXA-51_ genes were investigated by multiplex polymerase chain reaction (PCR). The blaNDM-1 gene was determined by simplex PCR.

**Results:**

fifty-eight strains were resistant to imipenem (98%), the modified Hodge test (MHT) was positive for 58 strains (100%), 47 strains (82%) were found to be positive for MBL by the test of double-disk synergy (DDST), 58 strains (100%) were positive by E-test MBL. The OXA 51 gene was detected in all strains, and the OXA 23 gene was detected in 53 strains (91%). In addition, the MBL genes were not detected by genotypic methods.

**Conclusion:**

the OXA-23 and OXA-51 carbapenemases type are responsible for the resistance to carbapenems in A. baumannii resistant to carbapenems in our establishment. Resistance to carbapenems by MBL enzymes has been found by phenotypic tests, which must be confirmed by genotypic methods; and solicit other MBL genes.

## Introduction

The emergence and spread of multi-drug resistant Gram-negative bacterial infections constitute a significant public health problem worldwide [1]. *A. baumannii* is associated with hospital-acquired infections which include ventilator-associated pneumonia, bloodstream infections, meningitis, and urinary tract infections (UTIs) [2]. Furthermore, infections due to carbapenemase-producing *A. baumannii* were associated with alarming rates of mortality [3]. During the last decade, the multi-drug resistance of *A. baumannii* has been reported increasingly, probably as a result of the extensive use of antimicrobial agents [4]. Carbapenem resistance in this species is observed increasingly worldwide and constitutes a sentinel event for emerging antimicrobial resistance [4].

Enzymatic degradation by β-lactamases is the most prevalent mechanism of β-lactam resistance in *A. baumannii*. Serine oxacillinases (ambler class D OXA-type) and metallo-β-lactamases (MBLs) (ambler class B) are β-lactamases with carbapenemase activity [5]. In the last years, MBL producing carbapenem-resistant strains of *A. baumannii* have been reported worldwide, without forgetting, oxacillinases carbapenemase-producing strains were detected widely [6]. Also, three types of metal beta-lactamases most described in *A. baumannii* strains, including imipenemase (IMP), verona imipenemase (VIM), and New Delhi metallo beta-lactamase (NDM)-1-types throughout the world [7].

In Morocco and especially in our region there is a lack of reports on the molecular basis of resistance in *A. baumannii*. In this study, the presence of MBL was investigated by phenotypic methods, and the responsible resistance genes were investigated by polymerase chain reaction (PCR) in *A. baumannii* strains, isolated from clinical isolates CHU Fez.

## Methods

**Bacterial isolated, identification, and antibiotic sensitivity testing:** this study analyzed 58 clinical isolates of *A. baumannii* resistant to carbapenems, not duplicated, isolated from diagnostic samples in bacteriology laboratory, from the various hospital departments of the Fez Hospital Center between November 2018 and June 2019. Isolation of all isolates of *A. baumannii* was performed using blood agar and bromocresol purple lactose agar. Identification was carried out using routine bacteriological tests based on morphological, cultural, and biochemical characteristics (Gram stain, API 20NE) and the Phoenix 100 Dickinson gallery. The strains were stored at -20°C in the Bacteriology Laboratory of the Fez Hospital Center. Only strains whose antibiogram shows resistance to imipenem are included in this study. Antimicrobial susceptibility testing was performed using the disc diffusion method and interpreted according to the Clinical and Laboratory Standards Institute (CLSI) recommendations [8].

**Modified Hodge test:** a 0.5 McFarland dilution of *Escherichia coli* American type culture collection (ATCC) 25922 was prepared. A 1: 10 dilution was streaked as a lawn onto a Mueller Hinton Agar (MHA) plate (Merck, Darmstadt, Germany) and a 10μg imipenem disk was placed in the center of the plate. *A. baumannii* strains were streaked in a straight line from the edge of the disk to the edge of the plate. After 24 hours, if test organisms had carbapenems, the test showed a clover leaf-like indentation of the *E. coli* growing along with the test organism growth streak within the disk diffusion zone [9].

**Double-disk synergy test DDST:** DDST was used for the phenotypic detection of MBLs in carbapenem-resistant Gram-negative bacteria, according to Lee *et al*. [9]. An ethylenediamine tetraacetic acid (EDTA) solution of 0.5 M concentration was prepared by dissolving 46.53 g of disodium EDTA-2H2O in 250 mL of distilled water and adjusting it to pH 8.0 by using NaOH. The mixture was sterilized by autoclaving. Two 10μg imipenem disks were placed on MH agar, and 4μL of an EDTA solution was added to one of them to obtain the desired concentration. The inhibition zones of the Imipenem and imipenem-EDTA disks were compared after 16 to 18 hours of incubation at 35°C. The increased inhibition zone ≥7 mm with the imipenem-EDTA disk was compared to the imipenem disk alone and was considered as MBL positive [9,10].

**MBL E-test:** the MBL E-test strip (bio-Merieux, Solna, Sweden) containing a double-sided of Imipenem (4 to 256 μg/ml) and Imipenem (1 to 64 μg/ml) in combination with a fixed concentration of EDTA was used for MBL detection. It was evaluated according to the instructions. MIC ratio of ?8 for the 2 reagent sides of imipenem and imipenem with EDTA was indicative of MBL production. This test was realized in all strains [11].

**Genotypic determination of carbapenemases production:** all the strains studied are inoculated on a medium of MHA at 37°C between 16 to 18 hours. Three to 4 colonies are suspended in sterile water 500μL and homogenized by vortex and then put in a water bath at 100°C for 10 min and put back on the ice for 2 min, then centrifuge for 10 min at 14,000 rpm, the supernatant was served at PCR. We used for PCR the Veriti thermal cycler and specific primers (Table 1). Responsible resistance genes were tested by PCR simplex and multiplex PCR in acinetobacter strains. The MBL bla_IMP_, bla_VIM_, and bla_NDM_ genes were investigated by simplex PCR. The bla_OXA-23_, bla_OXA-51_, genes were investigated by multiplex PCR [12-14]. The amplification reactions to detect MBL encoding genes performed in a volume of 50 μL containing, 2 μL of DNA template, 2.5 mM MgCl2, 0.4 μM of each forward and reverse primers, 100 μM of each dNTP, and 2 units of DNA polymerase (Promega, Madison, USA) in 1X PCR buffer provided by the manufacturer's instructions. The amplification conditions were described previously [13]. Then, three single (NDM, VIM, and IMP) PCR at 94°C for 5 min was programmed, followed by 35 cycles of amplification. Each cycle consisted of 95°C for 30 s, 58°C for 30 s, 72°C for 30 s. A final extension step (72°C for 10 min) completed the amplification [13,14]. PCR products were detected on 1.5% agarose gel (FMC Bioproduct, Rockland, ME) after ethidium bromide staining, UV illumination, and photographed by an Olympus digital camera (Olympus Soft Imaging Solutions GmbH, Munster, Germany).

**Table 1:** the primers of target genes used for PCR

Genes	Primer	Primer sequence (5'- 3')	TSA (Pb)
NDM	NDM-F	GGTTTGGCGATCTGGTTTTC	621
	NDM-R	CGGAATGGCTCATCACGATC	
IMP	IMP-F	GGAATAGAGTGGCTTAAYTCTC	232
	IMP-R	GGTTTAAYAAAACAACCACC	
VIM	VIM-F	GATGGTGTTTGGTCGCATA	390
	VIM-R	CGAATGCGCAGCACCAG	
OXA-51	OXA 51-F	TAA TGC TTT GAT CGG CCT TG	353
	OXA 51-R	TGG ATT GCA CTT CAT CTT GG	
OXA-23	OXA 23 F	GATCGGATTGGAGAACCAGA	501
	OXA 23 R	ATTCTTGACCGCATTTCCAT	

**Ethical approval:** this study was approved by the Joint Research Ethics Committee of Medical School and University Hospital Hassan II of Fez (Fez, Morocco).

## Results

**Bacterial isolates:** out of a total of 59 strains of *A. baumannii* isolates, 58 (98%) were resistant to imipenem. Among these strains, 23 (39.6%) were recovered from distal bronchial levy protected, 13 (22.4%) from central catheter, 11 (19%) from blood cultures, 6 (10.3%) from pus, 3 (5%) from urine, 2 (3.5%) from cerebrospinal fluid. The majority of *A. baumannii* isolates were collected from medical intensive care units (ICU) 47 (81%).

**Antibiotic susceptibility testing:** the antibiotic susceptibility results of the isolates are shown in Table 2. Susceptibility testing showed a high profile of resistance to the majority of antibiotics tested. In addition, among our isolates, fifty-eight (58) were carbapenem-resistant. Among these bacteria, no strains were detected resistant to colistin.

**Table 2:** antibiotic susceptibility of *A. baumannii* isolates from clinical samples

Resistance rates n (%)	
Antibiotics	Isolates (n= 59)
Piperacillin	59 (100%)
Ticarcillin	59 (100%)
Piperacillin/tazobactam	59 (100%)
Ticarcillin/clavulanic acid	59 (100%)
Ceftazidime	59 (20%)
Cefepime	59 (100%)
Imipenem	58 (94.3%)
Colistine	0
Tobramycin	59 (100%)
Amikacine	59 (20%)
Ciprofloxacin	59 (100%)
Gentamicin	59 (100%)

**Phenotypic detection of carbapenemases:** the MHT shows that all strains 58 (100%) produced carbapenemases. Moreover, the positivity of MBL was (82%) by DDST. In addition, all strains (100%) were found to be positive for MBL by the E-test (Table 3).

**Table 3:** positivity and negativity rates of MBL by phenotypic and genotypic tests

Phenotypic tests	MBL (+)	MBL (-)
DDST	47 (82%)	10 (18%)
MHT	57 (100%)	0
MBL E test	57 (100%)	0
Genotypic tests	(+)	(-)
MBL PCR	0	57
OXA PCR	57	0
Oxa 51	58 (100%)	
Oxa 23	53 (91%)	

**Figure 1:**
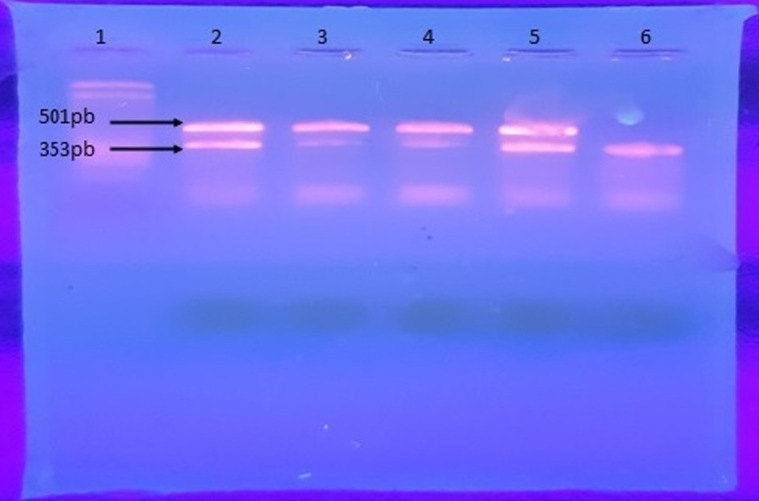
multiplex PCR gel showing of bla_OXA-23_ (501pb) and bla_OXA-51_ (353pb) positive acinetobacter strains; 1: ladder (100bp); 2: positive control: 3, 4, 5 and 6: clinical sample

**Genotypic identification of carbapenemases:** all strains were tested for oxacillinases and MBL genes by PCR and multiplex PCR. The bla_OXA-51_ gene has been proven in all strains 58 (100%), bla_OXA-23_ (90%). Furthermore, the MBL genes; including bla_IMP_, bla_VIM_ and bla_NDM_ were not detected (Figure 1).

## Discussion

*Acinetobacter baumannii* is an important opportunistic bacterial pathogen responsible for serious infections especially in intensive care units (ICU). In this study, most of the strains of *A. baumannii* were collected from ICUs (81%). *A. baumannii* has acquired a huge amount of resistance genes through horizontal transfers which makes it virulent and resistant to all environmental pressures [15]. Excessive use of antibiotics, especially carbapenems, contributes to the emergence of resistance in *A. baumannii*. In our study, antibiotic susceptibility testing showed that all isolates were resistant to most antibiotics, except colistin, and a high rate of resistance of imipenem (94%). This very high rate is alarming for our establishment, and it is similar to what was recently been reported in Morocco by Uwingabiye *et al*.2017 [16]. Outbreaks of *A. baumannii* multi-drug resistant (MDR) have been reported worldwide, including in North Africa [17,18]. The DDST showed a prevalence of resistance by MBL enzymes of 82%, this rate remains very high compared to other Moroccan studies, in particular, those carried out in Rabat in 2010, which reported a prevalence of 75% [18]. However, this prevalence rate is consistent with other studies worldwide, notably in Iran (62%) [7], Italy (62.5%) [19], China (55.6%) [20], in Turkey (53.7% in 2008 against 85.7% in 2015) [6,21] and in Pakistan 78% [22]. These results show that the frequency of these strains is increasing alarmingly all over the world. Their emergence represents a serious epidemiological risk for at least two reasons, on the one hand, these MBLs confer not only resistance to carbapenems but all beta-lactams and other classes of asphalt treated base (ATB); on the other hand, the genes encoding these enzymes are plasmid genes and can be transmitted horizontally to other bacterial strains.

Genotypic search for the genes encoding bla_IMP_, bla_VIM_, and bla_NDM_ which are the most described in *A. baumannii*, was negative in all strains. It was thought that EDTA could be responsible for the false positivity of MBL in phenotypic tests. Other studies have shown that the permeabilizing effect of EDTA on the membrane can increase the sensitivity of GNB, such as *A. baumannii* [23,24]. In contrast, these results may be true positives associated with another MBL gene that has not been screened in this study. Different studies have reported this difference between phenotypic tests and PCR, particularly in Turkey, Southern Hungary, Brazil, and China [25,26]. For this reason, carbapenem resistance genes, common in the region, should be investigated to properly assess the results of phenotypic tests. These results indicate that the options available for the appropriate treatment of infections caused by multidrug-resistant *Acinetobacter baumannii* are currently limited.

Although no MBL resistance gene was detected, the bla_OXA-51_ gene was isolated from all strains of *A. baumannii*, the bla_OXA-23_ gene from 90% of the strains tested, which appears to be responsible for the resistance to carbapenems in our hospital. Strains of *A. baumannii* type bla_OXA-23_ and bla_OXA-51_ producing enzymes have already been reported in Morocco [17]. Other studies report that strains of *A. baumannii* producing bla_OXA-51_ and bla_OXA-23_ type carbapenemases were detected, but no MBL gene (bla_IMP_, bla_VIM_, and bla_NDM_) [6,27]. The existence of bla_OXA-23_ and in the majority of strains, indicating the predominance of these genes among *A. baumannii* Moroccan strains. The emergence of bla_OXA-23_ in Morocco is compatible with the global epidemiology of bla_OXA-23_ and with many reports from Mediterranean countries [28]. We can deduce that the bla_OXA-23_ type carbapenemase was mainly responsible for the resistance of carbapenems in acinetobacter strains in our hospital. Resistance to MBL detected by phenotyping tests should be confirmed by genotypic methods. Currently, in vitro studies show that tigecycline and colistin are the only antibacterial agents with consistent activity against MBL producing strains [29].

## Conclusion

The prevalence of *A. baumannii* resistance to carbapenems is increasing in our hospital center. The production of MBL has been studied in phenotypic assays but has not been confirmed by PCR, which suggests that there are other MBL genes to be explored. PCR shows the dominance of the OXA23 gene. For this reason, it is necessary to monitor these strains to avoid their dissemination.

**Funding:** the study is part of a doctoral project approved and funded by the Center for Doctoral Studies at the University of Fez.

### What is known about this topic

- Carbapenem resistance is an emerging problem, and it has been reported worldwide;
- The prevalence of A. baumannii resistance to carbapenems has increased considerably in recent years due to the inappropriate use of antibiotics several other factors;
- In Morocco, A. baumannii resistant to carbapenems does not respond to any treatment except colimycine.

### What this study adds

- This study demonstrates the seriousness of the problem of resistance to carbapenems in A. baumannii in our establishment;
- The molecular characterization of A. baumannii is a first in our establishment;
- This study will enrich the Moroccan database concerning the problem of resistance to carbapenems.
